# Morphometry of the Scapular Notch and Its Clinical Implication in Suprascapular Nerve Entrapment

**DOI:** 10.3390/diagnostics15030346

**Published:** 2025-02-02

**Authors:** Jhonatan Duque-Colorado, Oscar Andrés Alzate-Mejia, Mariano del Sol

**Affiliations:** 1 Programa de Doctorado en Ciencias Morfológicas, Facultad de Medicina, Universidad de La Frontera, Temuco 4780000, Chile; 2 Centro de Excelencia en Estudios Morfológicos y Quirúrgicos (CEMyQ), Facultad de Medicina, Universidad de La Frontera, Temuco 4780000, Chile; 3 Departamento de Ciencias Básicas Biológicas, Universidad Autónoma de Manizales, Manizales 170001, Colombia; oalzate@autonoma.edu.co; 4 Facultad de Ciencias de la Salud, Universidad de Manizales, Manizales 170001, Colombia

**Keywords:** advanced anatomy, morphometry, scapula, scapular notch, neuropathy, suprascapular nerve

## Abstract

**Background/Objectives**: The aim of the present study was to evaluate the relationship between the type of scapular notch (SN), the morphometry of the SN, and the area of the suprascapular nerve (SSN). In addition to determining whether scapular notches other than Type VI, according to the classification of Rengachary, can generate a predisposition to SSN entrapment neuropathy. **Methods**: One hundred and sixty-nine dry scapulae were examined, the scapular notches were classified, according to the classification of Rengachary, and for each SN, the superior transverse diameter (STD), longitudinal diameter (LD), and area of the SN were determined. The SSN was dissected in five shoulders and its area was calculated. The data were analyzed in the statistical software SPSS. **Results**: The values for the STD, LD, and area of the SN showed significant differences between the types of scapular notches (*p* < 0.0001). Along the same lines, a considerable positive correlation (r = 0.79; *p* < 0.0001) was established between the area of the SN and the STD. Similarly, a very strong positive correlation (r = 0.87; *p* < 0.0001) was established between the area of the SN and the LD. This indicated that, as the STD and the LD increase, the area of the SN increases. **Conclusions**: Although different studies have reported an association between SN Type VI and the compression of the SSN by the formation of a bony hole that reduces the area of the notch, we have found that SN Type IV presented a smaller area among the types of notches and a smaller area than the SSN, which exposes the SSN to be closer to or in contact with the superior transverse ligament of the scapula, potentially subjecting the nerve to greater pressure and potentially resulting in SSN entrapment. This is evidence that should be considered in the clinical diagnosis of patients with entrapment neuropathy, since the type of SN and the area of the SSN can be determined by ultrasound, which contributes to a more accurate preoperative evaluation and diagnosis.

## 1. Introduction

The suprascapular nerve (SSN) is a mixed nerve originating from the upper trunk of the brachial plexus, which crosses the scapular notch (SN) below the superior transverse scapular ligament (STSL) to move towards the supraspinous fossa. It then curves around the spinoglenoid notch to move towards the infraspinatus fossa, innervating the infraspinatus muscle and providing sensory branches to the glenohumeral and acromioclavicular joints [1].

The SSN is susceptible to entrapment neuropathy, a condition that accounts for approximately 16% of all musculoskeletal complaints [2,3]. Factors that may give rise to SSN entrapment include the shape and size of the SN [4], the shape of the STSL [5], hypertrophy of the supraspinatus muscle [6], or the presence of the spinoglenoid ligament [7]. However, the shape and size of the SN have been identified as the most important elements in the etiology of SSN entrapment [8,9], as it is the most common site of compression and injury to the nerve along its course [10]. Therefore, this notch is considered one of the scapula’s most clinically relevant bony landmarks [4,11,12,13].

Given the clinical implications of the SN shape and size, Rengachary et al. [4] established a classification to characterize its variants. Within the six categories included in this classification, different studies have associated SN Type VI with SSN entrapment, because the STSL is ossified in this morphotype of the SN, forming a bony foramen, which consequently reduces the area of the SN and compresses the nerve [5,14,15,16].

Accordingly, the present study aimed to assess the relationship between the type of SN, according to the classification of Rengachary et al. [4], the SN morphometry, and the area of the SSN. This seeks to determine whether other types of scapular notches predispose individuals to suprascapular nerve entrapment neuropathy.

## 2. Materials and Methods

A descriptive–correlational, quantitative, non-experimental, cross-sectional study was conducted. The methodological planning of this study adhered to the guidelines established in the AQUA guide for conducting original anatomical studies [17].

To achieve the study objective, 169 dry scapulae from adult individuals were examined between March and July 2024. Of these, 47 were Chilean and obtained from the Anatomy Osteotheque in the Faculty of Medicine at the Universidad de La Frontera, Chile. The remaining 122 scapulae belonged to Colombian individuals and were obtained from osteotheques in the anatomy laboratories of the Universidad de Caldas, Universidad Autónoma de Manizales, and Universidad de Manizales, Colombia. For the scapulae to be included in the analysis, they had to meet the following two eligibility criteria: the base of the coracoid process and the margins of the SN had to be complete.

### 2.1. Classification of Scapular Notches

**Figure 1:**
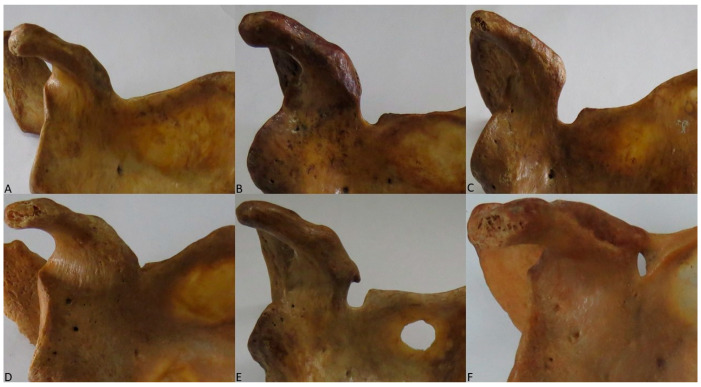
Types of scapular notches as classified by Rengachary et al. [4]. (**A**) Type I. (**B**) Type II. (**C**) Type III. (**D**) Type IV. (**E**) Type V. (**F**) Type VI.

Scapular notches were classified into 6 types, as categorized by Rengachary et al. [4]. Type I: wide depression from the superior margin of the scapula, from the superior angle of the scapula to the base of the coracoid process; Type II: wide, V-shaped notch with blunt tip; Type III: symmetrical, U-shaped notch with parallel margins; Type IV: shallow, V-shaped notch, the shallowness representing the bony impression of the SSN; Type V: notch with partial ossification of the medial area of the superior transverse scapular ligament; and Type VI: bony foramen inferomedial to the base of the coracoid process as a cause of the total ossification of the STSL (Figure 1).

### 2.2. Morphometric Analysis of Scapular Notches

**Figure 2:**
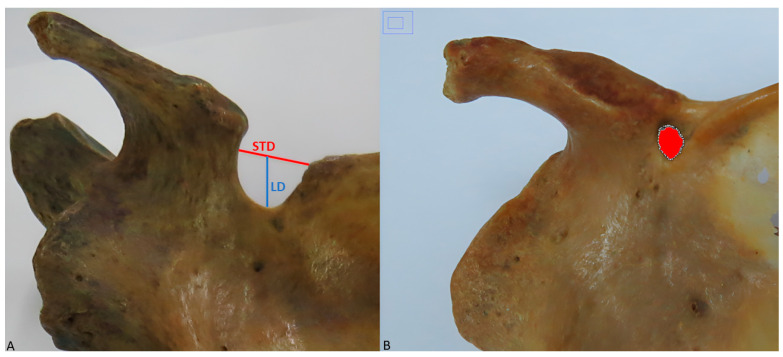
Morphometric measurements. (**A**) Superior transverse diameter represented by the red line and longitudinal diameter represented by the blue line. (**B**) Area of the scapular notch, Represented by the red-highlighted section.

The following morphometric measurements were performed for each SN:

1. Superior transverse diameter (STD): maximum horizontal distance between the corners of the SN (Figure 2A).
2. Longitudinal diameter (LD): maximum length of the SN, from the upper limit of the SN to its deepest point (Figure 2A).
3. Area of the SN: maximum extension of the SN considering its limits (Figure 2B).

STD and LD measurements were performed twice by two researchers with a TOTAL TMT321501 digital caliper (TOTAL TOOLS CO., Stgo, Chile) to an accuracy of 0.01 mm. The area of the SN was calculated using the ImageJ 1.54g software (National Institutes of Health, Bethesda, MD, USA), and the calibration was performed between the SN and the photograph using previous STD and LD measurements. For this analysis, those scapulae that presented SN Type I were excluded, since, due to their characteristics, the STD was not defined [4], and this variable is key to determining both the LD and the area of the SN.

### 2.3. Suprascapular Nerve Processing

The SSN was dissected in five individuals to contrast their area with the area of the scapular notches. For SSN processing, samples were fixed in 4% buffered formalin for 24 h and then embedded in Paraplast Plus (Sigma-Aldrich Co., St. Louis, MO, USA). Transverse sections 5 µm thick were made and stained with toluidine blue. Samples were visualized and scanned using the TissueFAXS i PLUS Cytometer TissueGnostics Axio Observer 7 Carl Zeiss GmbH TissueFAXS System (TissueGnostics GmbH, Vienna, Austria) to obtain preview brightfield images with the EC Plan-Neofluar 2.5x/0.085 M27 objective [2.5x, Air]. Then, the file was analyzed in StrataQuest 7.1.1.138, where the image preprocessing included the separation of two colors and the configuration of a virtual channel to obtain a gray image from which the detection of the major cross-sectional diameter, minor cross-sectional diameter, and SSN area was programmed in the virtual gray channel input with a manual threshold value of 40.

### 2.4. Statistical Analysis

The results were recorded in a database generated in Microsoft Excel 2019 software (Microsoft, Redmond, WA, USA) and analyzed in the SPSS statistical software, version 26 (IBM Corp., Armonk, NY, USA). Measurements were expressed as mean (standard deviation, SD) or median for continuous variables, according to the distribution of the data. Categorical variables were expressed as absolute values (*n*) and percentages (%).

The normality of the data obtained for each variable was evaluated using the Kolmogorov–Smirnov test. The data were then analyzed using a one-factor ANOVA or Kruskal–Wallis one-way ANOVA, including the Bonferroni correction. Additionally, the correlation of the data was established through the Pearson or Spearman correlation coefficient test. For this, α = 0.05 was considered significant.

## 3. Results

Of 169 scapulae, 165 met the eligibility criteria, while 4 were excluded from the study. Of the 165 scapulae analyzed, 94 (57%) presented left laterality and 71 (43%) right laterality.

Considering the classification by Rengachary et al. [4], the most prevalent type of SN in the scapulae was Type I, followed by Type III. The SN types with the lowest prevalence were Types V and VI. The details of the distribution of this classification are shown in Table 1.

**Figure 3:**
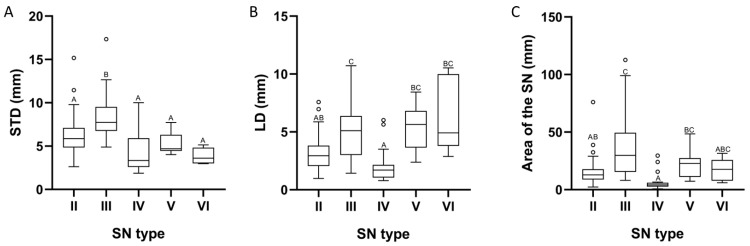
Graphical representation of the Kruskal–Wallis one-way ANOVA. The same letter in different scapular notch (SN) types means no significant differences. (**A**) SN type and superior transverse diameter (STD); (**B**) SN type and longitudinal diameter (LD); (**C**) SN type and area of the SN.

It was determined that the data analyzed did not approximate a normal distribution. Concerning the morphometric characteristics of the SN, STD measurements ranged from 1.87 mm to 17.34 mm, with a median of 6.15 mm. The STD values exhibited substantial disparities among the types of scapular notches (*p* < 0.0001), indicating that Types II, IV, V, and VI demonstrated uniformity in STD measurements, while differing from Type III (Figure 3A).

The LD values ranged from 0.79 mm to 10.72 mm, with a median of 3.51 mm. In the same vein, it was determined that SN Type IV presented a heterogeneous median in the LD compared to the Type III, V, and VI scapular notches. In contrast, the median in Type II showed differences only compared to Type III (Figure 3B). Thus, the LD of the scapular notches showed significant differences between types (*p* < 0.0001).

The area of the SN measurements ranged from 0.70 mm to 112.69 mm, with a median of 15.65 mm. The median area of the Type IV scapular notches exhibited variation compared to Types III and V, analogous to the relationship observed between Types II and III (Figure 3C). Thus, the medians corresponding to the area of the scapular notches presented significant differences among the notch types (*p* < 0.0001). Details for each of the morphometric variables evaluated are shown in Table 2.

**Figure 4:**
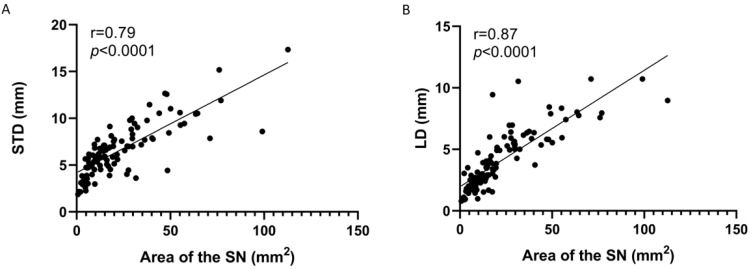
Correlation between measurements. (**A**) Area of the SN and superior transverse diameter (STD); (**B**) area of the SN and longitudinal diameter (LD).

The Spearman’s correlation coefficient test made it possible to determine a considerable positive correlation (r = 0.79; *p* < 0.0001) between the area of the SN and the STD. Similarly, a very strong positive correlation was established between the area of the SN and LD (r = 0.87; *p* < 0.0001). This indicated that, as STD and LD increase, the area of the SN increases (Figure 4).

**Figure 5:**
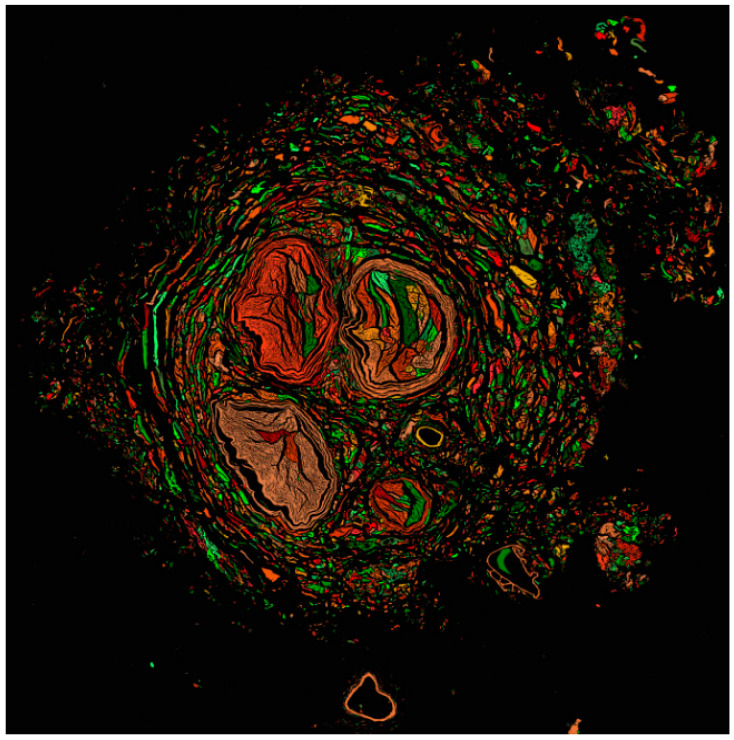
Detection of area by virtual gray channel at transverse section of the suprascapular nerve.

The analyzed sections of the SSN (Figure 5) revealed that, owing to its morphometric characteristics, it is a flattened nerve, exhibiting a larger transverse diameter of 3.11 ± 0.80 mm, smaller than all the values of the STD, a smaller transverse diameter of 2.97 ± 0.99 mm, which exceeds the LD of notch Types II and IV, and the area of 7.77 ± 0.28 mm, a value that exceeds the area of Type IV.

## 4. Discussion

The SN is a bony landmark in the scapula that provides information to safeguard the SSN in surgical procedures; in turn, the morphometric characteristics of the SN are a possible predisposing factor for SSN entrapment. Therefore, different methods have been established for its characterization. In the absence of a single method, our work used the classification by Rengachary et al. [4], as it allows the SN type to be easily identified from qualitative characteristics and is commonly used in the scientific literature. In the present study, we found a higher prevalence of scapulae with SN Type I (36.8%); whereas, the least prevalent morphotype was Type VI (4.8%), similar to the observation by Kaledzera et al. [18]. However, we found differences with other studies, where the highest prevalence was found in Type III and the lowest in Type I [4,19], discrepancies that could be due to the following two aspects: the origin of the study population, as established by other studies [20,21,22], and the age of the individuals, since it has been identified that partial or total ossification of the STSL arises as a consequence of aging, giving rise to SN Type V or VI, respectively [19,23], with Types I, II, III, or IV being in the early stages. Hence, we consider that there are differences in the results of our study, since age was an intermittent statistic in the different studies; furthermore, our samples correspond to Chilean and Colombian populations, which is different from that of other studies.

In our analysis of the morphometric characteristics of the SN, we evaluated two diameters, the STD and LD, similar to other works [20,21], and we omitted the mean transverse diameter (MTD), a parameter considered for the characterization of the SN in some studies [8,18]. We excluded the MTD, as we deem it an extraneous parameter encompassed by the STD, given that the MTD is never smaller than the STD in any type of SN; consequently, if the SN presents a transverse diameter much smaller than the STD, the morphometric parameters related to the transverse diameter of the SN will not pose a risk for injury or compression of the SSN.

Regarding the STD, we determined differences between the types of suprascapular notches (*p* < 0.0001), an aspect similar to that demonstrated by Albino et al. [20]; however, their study noted differences among all types of suprascapular notches, and the lowest values were associated with Type VI, while in our study, differences were found between two large groups: one group formed by Types II, IV, V, and VI and a second group by Type III, where the highest associated values were found. This indicates that an individual with scapulae with SN Type III will be at a lower risk of SSN compression due to the width of the SN. In contrast, individuals with Types II, IV, V, and VI will have a higher risk, considering that these notches are associated with lower STD values. As has been pointed out by Rengachary et al. [4] and Polguj et al. [9], the narrowing of a SN may predispose a patient to SSN entrapment neuropathy.

Regarding the LD, Sangman et al. [24] determined in 104 scapulae of Indian origin that the smallest measurements were present in SN Type IV, while the largest measurements were found in Type V scapulae. These results differ from those reported by [21], where Type V presented the shortest distances for the LD, and the longest distances were associated with Type III. Our results partially agree with the previously mentioned studies, since we found that the LD is a parameter dependent on the type of SN (*p* < 0.0001), where the shortest and longest LD are associated with Types IV and III, respectively. Consequently, according to our results, presenting SN Type IV positions the SSN closer to or in contact with the STSL, thereby increasing pressure on the nerve and possibly resulting in SSN entrapment [25].

This is supported by the data obtained regarding the area of the SN, where the smallest areas were associated with notch Types II, IV, and VI. Among them, Type IV presented the most critical value, which was to be expected, since its shape represents the impression of the SSN. Nevertheless, studies generally point out that the smallest areas for SN are associated with Type VI, considering it a risk factor for SSN compression [5,14,15,16], omitting the risk that other types of notches, such as Type IV, may pose. This risk is given by two characteristics that correlate positively and strongly (*p* < 0.0001) with the area of the SN, such as STD and LD, since, as the SN area is reduced, the STD and LD are reduced, which could increase the likelihood of SSN compression.

The morphometric characteristics of Type IV show that its area (4.24 mm) is smaller than that of the SSN (7.77 ± 0.28 mm), where the smaller transverse diameter (2.97 ± 0.99 mm) of the SSN is larger than the LD of the SN, so that, in individuals with scapulae with SN Type IV, the SSN will be in direct contact with the STSL. Consequently, the shoulder movement with crossed abduction or adduction will strain the nerve and cause its compression with the ligament, causing pain. The smaller transverse diameter of the SSN (3.11 ± 0.80 mm) did not exceed the STD values of the SN; however, this value in the SSN could be biased by the contraction of approximately 20% that the peripheral nervous system undergoes when fixed for more than 12 h [26,27]. In this sense, the SSN would probably exceed this dimension of the SN, the compression of which would not be relieved only by sectioning the STSL. The resection of the SN margins should also be performed to increase its size, as demonstrated by Rask [28] in two patients who experienced intense pain during protraction and where the transverse diameter of the SN was responsible for the compression neuropathy.

Clinical assessments such as the SSN stretch test are used to identify SSN compression neuropathy. This test is considered positive when it causes increased pain in the back of the shoulder. However, diagnosing this condition exclusively by clinical examination can be complex, since various shoulder pathologies present similar symptoms [29]. Thus, imaging is required to continue with its clinical diagnosis, making use of simple shoulder radiographs and CT scans, where the characteristics of the SN can be visualized [23] or even, in this case, making use of more complete and accessible tools, such as ultrasound, which, in addition to the morphometric characteristics of the SN, can identify the dimensions of the SSN. Therefore, we consider that these findings could acquire clinical importance by contributing to more accurate diagnoses involving the SSN and SN.

## 5. Conclusions

The morphometric characteristics of the SN are closely related to the type of SN. Although different studies have associated Type VI with SSN entrapment neuropathy, we have found that Type IV presents smaller dimensions than the SSN and any SN morphotype, making this type of notch a risk factor for the SSN. We believe that these findings contribute to a better understanding of the anatomy of the scapula and attain clinical importance by contributing to diagnoses involving the SSN.

It is essential to advance in imaging studies to identify those morphometric characteristics of the SN that represent a risk factor for the SSN.

## Figures and Tables

**Table 1:** Prevalence of scapular notch type.

SN Type	*n*	%
Type I	56	34.0
Type II	37	22.4
Type III	42	25.5
Type IV	17	10.3
Type V	8	4.8
Type VI	5	3.0
Total	165	100

SN, scapular notch; *n*, absolute value; %, percentage.

**Table 2:** Superior transverse diameter, longitudinal diameter, and scapular notch area, according to scapular notch type.

Parameter	Type II	Type III	Type IV	Type V	Type VI	Value *p*
STD	5.86	7.73	3.34	4.69	3.61	1.68 × 10^−8^
LD	2.94	5.11	1.71	5.64	4.92	1.17 × 10^−7^
Area of the SN	12.81	29.86	4.24	22.84	17.70	1.33 × 10^−8^

Values were expressed as the median. STD: superior transverse diameter; LD: longitudinal diameter; SN: scapular notch.

## Data Availability

The data presented in this study are available on request from the corresponding authors.
